# Investigating looking and social looking measures as an index of infant violation of expectation

**DOI:** 10.1111/desc.12452

**Published:** 2016-10-26

**Authors:** Kirsty Dunn, J. Gavin Bremner

**Affiliations:** ^1^ Department of Psychology Lancaster University UK

## Abstract

Accumulated looking time has been widely used to index violation of expectation (VoE) response in young infants. But there is controversy concerning the validity of this measure, with some interpreting infant looking behaviour in terms of perceptual preferences (Cohen & Marks, 2002; Haith, 1998). The current study aimed to compare the use of looking time with a recently used measure of social looking (Walden *et al*., 2007) in distinguishing between 6‐month‐old infants’ response to novelty/familiarity and a condition in which the object was covertly switched for a different object. Following habituation, infants showed more social looking in response to the object‐switch condition than the novel object change, whereas the more commonly used accumulated looking time measure did not distinguish between the two, showing an increase for both. Thus, social looking is a more valid measure of infant VoE than looking time.

## Research highlights

- This paper compares accumulated looking and social looking behaviour in response to perceptual novelty and VoE.
- Results reveal that social looking specifically indexes VoE, whereas accumulated looking indexes both VoE and novelty.
- It is proposed that social looking may provide an unambiguous behavioural measure of VoE in future work.

## Introduction

Following the observation that infants repeatedly familiarized to a visual stimulus preferentially look to a novel stimulus (Fantz, 1967), accumulated looking time has been used in habituation paradigms to indicate detection of perceptual novelty. The measure has been used successfully from birth onwards to investigate a number of topics including, but not limited to, form perception (Caron, Caron & Carlson, 1979; Day & McKenzie, 1981; Slater, Mattock, Brown & Bremner, 1991), numeracy (Antell & Keating, 1983; Xu & Spelke, 2000), and memory (Cohen, DeLoache & Pearl, 1977; Fagan, 1973; Pancratz & Cohen, 1970).

More recently, accumulated looking has been used to measure higher‐level understanding, the rationale being that longer looking at events that do not accord with principles of physical reality index violation of the infants’ expectations, and hence their understanding the principles in question. Such violation of expectation (VoE) tasks have been applied to, among other topics, infants’ knowledge of numeracy (Wynn, 1992; Xu & Carey, 1996) and object permanence (Ahmed & Ruffman, 1998; Baillargeon, 1987; Baillargeon & Graber, 1988; Leslie & Keeble, 1987). However, it is hard to separate VoE from lower‐level perceptual causes of longer looking. Through changing a number of parameters in their dynamic model, Schöner and Thelen (2006) demonstrated that infant looking behaviour can be driven by a number of methodological differences that are likely to have been overlooked by researchers, and these differences can easily have an influence on the balance of familiarity and novelty looking preferences. For example, the use of, and time exposed to, pre‐trials, commonly used in VoE experiments, to familiarize infants with outcomes before manipulations could lead some infants to respond on the basis of familiarity preferences (Hunter & Ames, 1988). Although VoE experiments have generally been designed to rule out perceptual novelty bases for looking experiments, some judge these attempts to be unsatisfactory. Taking this into account, Haith (1998) argued that cognitive interpretations of accumulated looking data in response to this paradigm lacked parsimony, particularly because at a perceptual level, both novelty and familiarity preferences may result in longer looking (Hunter & Ames, 1988; Schöner & Thelen, 2006). In addition, one might argue that aspects of novelty should not be removed from such experiments, as an event that violates a physical law should, in some sense, be novel. Thus it is vital to have a measure that specifically indexes VoE and not perceptual novelty or familiarity. This has led to the search for alternative measures of infant expectation, such as eye‐tracking methods (Bremner, Slater, Hayes, Mason, Murphey *et al*., under revision) and brain indices of error detection (Berger, Tzur & Posner, 2006) but these approaches have their own limitations.

Early emergence theories of social looking (Vaillant‐Molina & Bahrick, 2012; Walden & Ogan, 1988) support the measurement of this behaviour as a useful index of ambiguity from 6 months. Walden, Kim, McCoy and Karrass (2007) investigated social looking as a measure of infant VoE in Wynn's (1992) VoE numeracy task, measuring looks to the parent following correct and incorrect numerical outcomes.

Following baseline and pre‐test trials in which 6‐ and 9‐month‐old infants were familiarized to the Elmo puppets and their movements, addition and subtraction events were presented with both expected and unexpected numerical outcomes. Previous findings were replicated, as infants looked longer to incorrect stimulus outcomes than correct outcomes. In addition, infants engaged in more social looking towards the caregiver following incorrect outcomes. There were no significant age‐related differences reported for either measure, although descriptive data showed more social looking in the 9‐month‐old age group. This could easily be accounted for, though, by the seating position of the infant which required a challenging 180° turn upwards to view the caregiver, which may have been harder to execute for younger infants. This study, therefore, demonstrated that social looking increased following incorrect numerical outcomes. Social looking thus appears to be as reliable a measure of error detection in Wynn's task as accumulative looking. However, it has been argued that Wynn's (1992) looking time results could arise on the basis of familiarity rather than VoE due to the task procedure designed: numerically incorrect results were also familiar outcomes and so longer looking to the stage might have reflected a perceptual preference for familiarity rather than evidence of VoE (Cohen & Marks, 2002). On the face of it, social looking is also open to lower‐level perceptual interpretations. It might increase just because an event is novel or familiar. Thus this newer measure only has particular utility if it can be demonstrated to be sensitive to VoE but not to perceptual novelty/familiarity effects.

In order to see if social looking specifically indicates VoE, we measured social looking and conventional accumulated looking time in response to both perceptual novelty and a condition that violated expectations of object identity (object‐switch). The novelty condition involved an object change that did not violate physical principles, whereas the object‐switch condition involved an illegitimate object change. Note that in both conditions, there is an element of novelty introduced in the test trial. The experimental logic is that there is scope to measure a specific response to violation of expectation in an event that also contains novelty, as many VoE experiments do. Thus, should infants initiate social looking in both conditions, this would indicate that social looking is not a specific measure of VoE as there is an element of novelty in both. If infants selectively initiate social looks following the illegitimate object change, this would indicate that social looking indexes VoE (in this case object identity violation) rather than perceptual novelty detection alone.

## Method

### Participants

Twenty 6‐month‐old infants (10 girls, *M *=* *194.88, range = 171–213 days) were recruited through phone calls from a database compiled of those mothers who gave birth at the Royal Lancaster Infirmary and expressed an interest in taking part in psychological research. This sample size was chosen based on previous research using violation of expectancy where at least eight and commonly 10 participants per condition are reported (Feigenson, Carey & Spelke, 2002; Xu, Spelke & Goddard, 2005). Data collection concluded following successful habituation of 10 infants per condition. Prior to recruitment, ethical clearance regarding the recruitment, methodology, and data handling throughout the study was sought and gained. Data from an additional nine infants could not be used due to technical problems (one) or failure to habituate (eight).

### Materials

Events were presented on a stage, recorded and shown to infants on a television monitor (38 cm × 70 cm). The stage (91 cm wide and 192 cm high) was lit by a fluorescent tube located in the top recess, and had an opening (26 cm high and 66 cm wide) 13 cm from each side and 17 cm from the top of the stage. A rotating screen (66 cm wide and 26 cm high) was positioned in the centre of the stage. There was a large blind that could be lowered to fully hide the stage from the infants. There was an opening (22 cm wide, 58 cm from the rear of the stage, and 33 cm high) on the left‐hand side of the stage to allow experimenters to manipulate the objects. Objects were a hedgehog (12 cm × 10 cm) and a snail (12 cm × 10 cm), objects that were selected because another group of infants showed no preference for one over the other.

### Procedure

**Figure 1:**
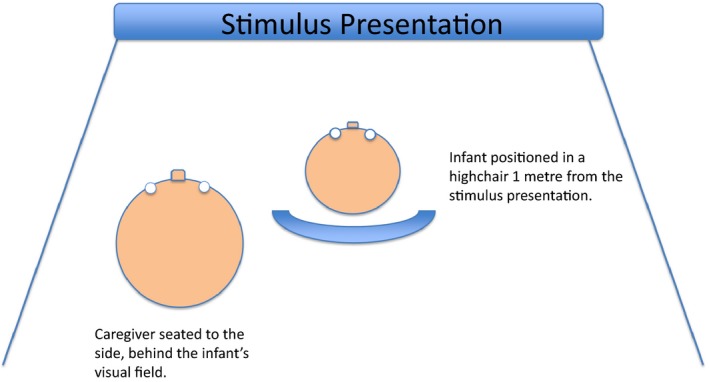
The experimental set up of stimulus presentation, infant and caregiver position.

Infants sat in a high chair at a viewing distance of 1 metre from the television screen. The parent taking part in the study sat slightly behind and to the side of the infant so that the parent was not in the eye‐line of the infant but only a small head‐turn was required for the infant to bring the caregiver into view (Figure 1.). In order to minimize visual distractions, the infant and caregiver were surrounded by matt black boarding and the high chair extended above the back of the infant's head. The task bears similarities to Wynn's (1992) task in terms of the hiding events shown to the infants. Infants were assigned to novelty or object‐switch conditions. Within these conditions, infants saw either the hedgehog or the snail as the habituation object. During habituation trials, infants witnessed a hand, holding the toy, enter the stage and place the toy behind the screen. The empty hand was then shown to the infant before exiting the stage. The screen was then lowered to reveal the toy. The trial was terminated after 30 seconds or if the infant looked away from the screen for more than 4 seconds. The attention‐getter in the form of a short video clip of an animated lobster was presented for 2 seconds between each trial to re‐establish infant attention. Habituation trials continued until the mean looking time to the screen during two consecutive trials reduced to 50% of the mean of the first two trials. Following this, the first of three test trials commenced. Infants in the novelty condition saw the hand enter the stage holding the other (novel) toy (the hedgehog if the habituation toy had been the snail, and vice versa) and placing it behind the screen. The empty hand was shown to the infant before exiting the stage. The screen was then lowered to reveal the same toy as was hidden. Infants in the switched‐toy condition saw the hand enter the stage holding the familiar toy from the habituation trials. This toy was placed behind the screen and the empty hand was shown to the infant before exiting the stage. The screen was then lowered to reveal the other, illegitimate, toy.

Caregivers were instructed only to interact with the infant if s/he initiated a social look or became distressed. Events were recorded for looking coding. Two primary types of infant looking behaviours were coded from the recordings by observers: duration of time spent looking to the stage (the previously used measure of interest or discrimination), and the number of infants’ social looks. The number, rather than duration, of social looks was chosen as a more appropriate measure of social checking behaviour rather than a measure of infant interest in the caregiver. Social looking constituted any look that would bring the caregiver into the infant's visual field. A screen prevented the infant from viewing anything else in this direction so that the target of the head turn could be clear. All infants were deemed physically capable of the head turn required in order to do this. Both measures were taken from the moment the screen was lowered to reveal the outcome and social looking was recorded once the infant had seen the outcome. A second investigator coded looking times and number of social looks from digital recordings of 25% of the sample to assess inter‐observer reliability. Interobserver correlations were high for accumulative looking time to the stimuli, Pearson *r *=* *0.90, *p *<* *.001, and number of social looks, Spearman's *r*_s_ = 0.81, *p *=* *.014.

## Results

Accumulated looking time and number of looks was compared using repeated‐measures analysis of variance (ANOVA) and independent‐measures *t*‐tests. Due to positive skew and the presence of zeros in the social looking counts, log transformation was applied to the accumulated looking time and log transformation +1 was applied to the looking data. An alpha value of 0.05 was used for statistical analysis.

### Looking time to the array

**Figure 2:**
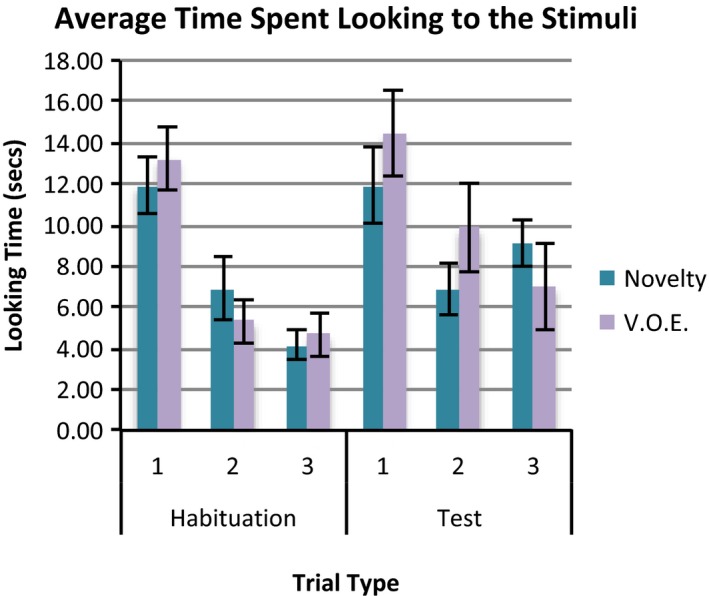
The average looking time to the stimuli on stage following habituation and test trials for infants in the novelty and VOE conditions. Bars represent standard error.

Figure 2 shows infant looking time to the stage on the habituation and test trials of the novelty and object‐switch conditions.

Based on the data for the final habituation trial and the first test trial, a repeated‐measures ANOVA with one within‐measure; trial type (habituation vs. test) and three between‐measures; condition (novelty vs. object‐switch), habituation stimulus (hedgehog vs. snail) and gender, revealed that infants looked to the stage for significantly longer following the first test than the last habituation trial, *F*(1, 18) = 31.35, *p *<* *.001, *η*_*p*_^2^ = 0.72. There were no other significant main effects or interactions.

Significant recovery of looking on test trials was found for those in both the novelty, *t*(9) = 3.19, *p *=* *.011, and object‐switch conditions, *t*(9) = 5.15, *p *=* *.001.

### Social looks analysis

**Figure 3:**
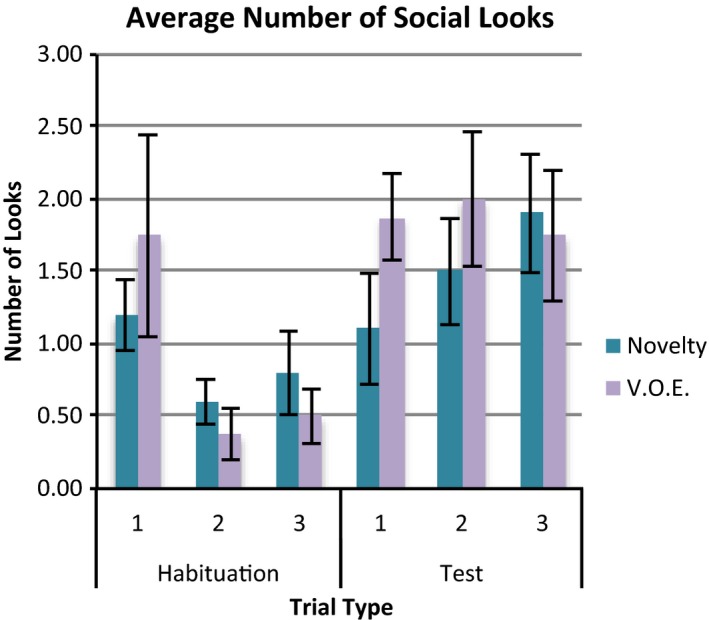
The average number of social looks initiated following habituation and test trials for infants in the novelty and VOE conditions. Bars represent standard error.

Figure 3 shows number of social looks on the habituation and test trials of the novelty and object‐switch conditions. Infants in the object‐switch condition showed more social looking on the first test trial than those in the novelty condition.

Based on the data for the final habituation trial and the first test trial, a repeated measures ANOVA with one‐within measure: trial type (habituation vs. test), and three between‐measures: condition (novelty vs. object‐switch), habituation stimulus (hedgehog vs. snail), and gender, revealed a significant effect of trial type, *F*(1, 18) = 19.49, *p *<* *.001, *η*_*p*_^2^ = 0.52. This was qualified by a significant interaction between trial type and condition, *F*(1, 18) = 9.89, *p *=* *.006, *η*_*p*_^2^ = 0.38. Pairwise post‐hoc analyses indicated that infants in the novelty condition did not show a significant difference in social looking between the final habituation and first test trial, *p *=* *.32. However, those in the object‐switch condition initiated significantly more social looks following the first test trial (*M *=* *1.88, *SD* = 0.82) than the final habituation trial (*M *=* *0.38, *SD* = 0.52), *p *<* *.001. There was no significant difference between conditions on final habituation trials, *p *=* *.28. However, on the first test trial, infants in the object‐switch condition initiated more social looks (*M *=* *1.88, *SD* = 0.82) than those in the novelty condition (*M *=* *1.1, *SD* = 1.2), *p *=* *.028.

Based on data from all three test trials, a repeated‐measures ANOVA with one within‐measure (test trial number 1, 2 and 3) and three between‐measures: condition (novelty vs. object‐switch), habituation stimulus (hedgehog vs. snail) and gender, revealed a significant interaction between trial number and condition, *F*(1, 18) = 3.37, *p *=* *.046, *η*_*p*_^2^ = 0.16. Post‐hoc pairwise comparisons showed no significant difference in social looking across test trials in either condition. However, those in the object‐switch condition initiated more social looking (*M *=* *1.88, *SD* = 0.82) than those in the novelty condition (*M *=* *1.1, *SD* = 1.2) on the first test trial only, *p *=* *.028.

## Discussion

Infants in both the novelty and object‐switch conditions looked longer to the stage on test trials than on habituation trials with no significant difference in looking to the stimuli between groups. Thus, recovery of looking occurs both following perceptual novelty and an illegitimate change of object identity and this indicates that this measure does not differentiate between VoE and perceptual novelty. It is of course possible in this case that infants in the object‐switch condition responded to the novelty of the revealed object rather than the violation of the identity change. Although well‐designed VoE experiments control for perceptual novelty, it is not always possible to eliminate low‐level factors that might lead to a novelty preference, and research has shown that familiarity preferences can occur under certain circumstances (Hunter & Ames, 1988; Schöner & Thelen, 2006), leading to a perceptual familiarity interpretation of Wynn's (1992) work. Adding to these interpretative concerns, we have demonstrated a task setting in which the standard looking time measure does not distinguish between perceptual novelty and VoE. Considering the range of research using preferential looking to measure infant knowledge, the implications of this are widespread.

In contrast, infants in the object‐switch group initiated more social looks during the first test trial than the final habituation trial, whereas there was no such effect in the novelty condition. The object‐switch condition contained an element of novelty in addition to violation of expectation and so it is possible that this is the result of response to novelty. However, as both types of test trial contained equal elements of novelty, this could not easily explain increased social looking in the object‐switch trial only. Thus our preferred interpretation is that the increase in social looking in the object‐switch condition signalled a response to VoE over and above the element of perceptual novelty. In our view, this further strengthens the utility of the social looking measure, indicating that it is sensitive to VoE even when perceptual novelty exists. In short, this measure distinguishes between perceptual novelty and VoE and so is an unambiguous measure of VoE. As such, this is the first study to find a behavioural measure that specifically indexes VoE.

Further research should extend work to investigation of social looking in response to other VoE events including those that do and do not contain an element of novelty. Social looking could be used to clarify preferential looking data in several domains of infancy research. For example, Baillargeon, Spelke and Wasserman (1985) habituated 5‐month‐old infants to a drawbridge rotating through 180° towards and away from the infant. On test trials, a box was placed behind the rotating screen, which then either stopped rotating at the box (possible) or continued to rotate to the floor as in the habituation trials (impossible). Infants looked longer to the impossible event and this has been interpreted as evidence for an understanding that the screen should not be able to pass through the box, and thus an understanding of object permanence. However, Rivera, Wakeley and Langler (1999) presented evidence indicating that longer looking to the impossible event could be due to perceptual differences between the two test conditions, specifically a preference for more motion. The measurement of social looking in response to tasks such as these could clarify whether infants are responding specifically to violation of their expectation of the location of the object or to some other lower‐level stimulus property.

The fact that 6‐month‐old infants showed reliable, appropriate social looking in response to incorrect outcomes provides some evidence for at least a precursor of social referencing in early infancy. This shows some support for early emergence theories of social referencing (Vaillant‐Molina & Bahrick, 2012; Walden & Ogan, 1988) in contrast to Schaffer's (1989) topic sharing theory that does not predict meaningful social looking behaviour until 12 months of age.

In conclusion, this study supports the need to be cautious when interpreting preferential looking data. Although infants could be responding on the basis of VoE, it has been shown that in the present task this measure does not distinguish between VoE and perceptual novelty/familiarity preference. In contrast, social looking emerged as a specific measure of VoE, and thus this measure may provide an unambiguous measure of VoE in future work.
